# Tubercular Pneumonia

**Published:** 1871-05

**Authors:** D. Francis Condie

**Affiliations:** Philadelphia


*Tubercular Pneumonia.* By D. FRANCIS CONDIE, M. D., of Philadelphia.

[From American Journal of the Medical Sciences.]

Notwithstanding that everything relative to the etiology, pathology, pathognomonic symptoms, therapeutic and hygienic management of pulmonary tuberculosis, has been subjected to the most careful investigation by some of the most reliable observers, whose researches have been fully laid before the profession, still it seems to us that a very large number of physicians, even some distinguished teachers, have entirely overlooked the fact that, for the protection of *phthisis pulmonalis*, the mere deposit of tubercles in the tissues of the lungs will not suffice, but that in every case there must be the concurrence of an error of the nutrition—tuberculization—on the one hand, and, on the other, an inflammation, usually chronic or subacute in character, though in many cases eminently acute.

That the disease known as pulmonary phthisis is not, as is so generally supposed, the result solely of the presence of tubercles in the lungs, all, we believe, will be convinced by a careful study of its pathognomonic symptoms, its progress, and its most common termination, with an investigation of the lesions discovered upon an examination of the lungs after death. In many instances, where death has taken place suddenly from various appreciable causes, we have detected the existence of tuberculization of the lungs; occasionally, to some considerable extent, where not a single symptom had been noticed during the lifetime of the patient indicative of the presence of what would be denominated, in common parlance, "confirmed consumption," or, indeed, of any common pulmonary disease.

Pulmonary consumption (*phthisis pulmonalis*), in the strict acceptation of the term, is never developed with all its pathognomonic symptoms until some grade of inflammation is set up in a lung which is already the seat of tuberculization. In many of the cases inflammation plays so predominant a part that the name *tubercular pneumonia* might, with great propriety, be applied to them. In such cases, the disease very generally runs its course to a fatal termination with great rapidity.

Tubercles, when deposited in the lungs to any extent, give rise usually, sooner or later, to a marked disturbance of the healthy play of many of the functions of the living economy, superadded to that defect of nutrition throughout every portion of the body, incident always to the peculiar diathesis to which tuberculization appertains; there is more or less dyspnœa, especially when exercise is taken, a short dry cough, a dry skin, susceptibility to chilliness, hectic fever, generally a small, rapid, feeble pulse, night sweats, progressive and finally extreme emaciation, colliquative diarrhœa, terminating—after a more or less protracted course—in death.

Whenever the deposition of tuberculous matter occurs slowly, and dispersed throughout a large portion of the substance of the lungs, and the patient is able to take an adequate amount of appropriate food, and is constantly surrounded by favorable hygienic conditions, among which stands prominently a dry, temperate, equable atmosphere, and daily active exercise in the open air, symptoms of disease of a serious character are comparatively long in presenting themselves; almost invariably the case follows a slow, insidious course, and, with a slowly increasing exhaustion of the physical and vital powers of the system, the patient lingers on, year after year, before death takes place.

When at any one portion of either of the lungs, or both, a large number of tubercles are deposited in close proximity to each other, they, sooner or later, acting as foreign bodies, produce a low grade of inflammation in the thin layer of lung tissue interposed between the individual tubercles, which becomes, in consequence, gradually destroyed. The mass of tubercular matter, thus formed by the aggregation of the separate tubercles, becomes, after a time, softened and disintegrated; and if a large bronchial ramification passes in its immediate vicinity, a communication between the cavity of vomica and the bronchi may be formed, attended with an expectoration of a puruloid fluid, mixed with particles, more or less numerous, of a cheese-like appearance. In this manner the entire mass of tubercular matter may be discharged from the lung, and when the other portions of the organ are free, or very nearly so, from tubercles, and the patient at the same time is placed under favorable hygienic circumstances, the sides of the vomica, left by the discharge of the softened tuberculous matter, gradually coalesce and form a kind of cicatrix. In such cases, which we have reason to believe are of more frequent occurrence than is generally supposed—we have met with several in our own practice—there may take place a return to a comfortable or even entire state of health. In, however, the majority of cases of tuberculous disease of the lungs, the deposition of tubercles, varying in size at different points, occurs in patches of greater or less extent, disseminated throughout a large portion of one or both lungs. These run the same course as above described, in reference to the larger and more circumscribed deposits of tubercular matter, and form numerous separate vomicæ; but few of which, however, having any outlet for the discharge of their contents, their presence excites an irritation of the surrounding tissues, which passes over ultimately into subacute inflammation, attended with a constant cough, copious expectoration, hectic fever, night sweats, &c. The vitality of the system is thus gradually exhausted, and finally ceases. This is the condition of things which is met with in the majority of the chronic cases of pulmonary consumption.

When, however, a patient affected with tuberculosis is, from exposure to cold and damp, or to sudden transitions of atmospheric temperature, or from deficient clothing, attacked with bronchitis or pneumonia, the course of the disease towards a fatal termination is very rapid, as well on account of the crippled condition of the tuberculated lung, rendering it incapable of sustaining the inflammatory process set up in its tissues until a favorable close occurs, as from the depressed vitality of the entire system, which is invariably attendant upon tuberculosis, precluding a resort to the therapeutic means adapted to arrest the inflammation of the lung tissues previously to their entire disorganization.

To this latter class of cases we believe the term "*tubercular pneumonia*" is most appropriate. Thus named, we are convinced that it would lead to a more correct conception of their true pathology—their recognition as cases of inflammation seated in the tissues of a tuberculosed lung—an inflammation which has been excited by causes altogether independent of, though modified in its character, march, and termination, by the tubercular condition of the tissues in which it is seated. Inflammation, when occurring in other tuberculosed tissues, has already been recognized in medical nomenclature as tubercal, a phrase which, though not absolutely correct, is useful as pointing out an important fact to be kept in view in forming our diagnosis and determining our plan of treatment. The profession almost universally recognize the convenience of the terms tubercular meningitis, tubercular peritonitis, &c.; why not also, then, tubercular pneumonia? indicating, respectively, inflammation seated in the tuberculosed meninges of the brain, in a tuberculosed peritoneum, in tubercular lungs, &c.

Every practitioner must have met, as we have repeatedly, with cases in which the deposition of tubercles in the tissues of the lungs had been going on for some time unsuspected by the patient or his friends—the slight cough, the progressive emaciation, the gradual reduction of strength, the slight afternoon exacerbation of fever, with circumscribed coloration of the cheeks, and the other indications of slowly declining health, being referred to causes altogether irrespective of the serious mischief going on in the lungs, until, from exposure on the part of the patient to a cold and damp atmosphere when inadequately clothed, to a sudden, unexpected change from a dry, mild state of the weather to one chilly and damp, or from any other cause, he becomes attacked with bronchitis or pneumonia, all the pathognomonic symptoms of what is popularly denominated "confirmed consumption" become, with great rapidity, developed. It is this circumstance—pneumonia occurring in subjects affected with pulmonary tubercles—that has led certain pathologists into the error of classing tubercular deposits in the lungs among the results of inflammation when it attacks those of a strumous diathesis. In this error they believe themselves to be confirmed by the fact that, in individuals predisposed to pulmonary tuberculosis, in whom inflammation of the lungs occurs, which in such patients almost invariably assumes a subacute or chronic form, tubercles are liable to be developed in the lungs either during the protracted course of the inflammation, or as its sequela, particularly if the inflammation is mismanaged hygienically or therapeutically.

The correctness of the views advanced in this paper we have satisfied ourselves of by the facts developed during a somewhat extended series of clinical observations, and they are, in our estimation, of a highly important character, inasmuch as they lead to a correct estimate of the true prophylaxis in those cases in which there is reason to believe that the occurrence of pulmonary tuberculization is to be apprehended or has already taken place. It must be evident that, in such cases, to insure the patient's safety, all those causes which have a tendency to produce inflammation of the lungs must be carefully avoided. Whenever circumstances will permit, the most prudent course will be the removal of the patient from a damp, changeable climate to an inland location, where the air is dry, pure, and of an equable and moderate temperature. The patient should avoid confinement in close, overheated apartments, or sedentary occupations in those which are damp and chilly. On the contrary, he should be urged to take daily active exercise in the open air, on foot or on horseback, properly clothed. He should occupy, as sleeping apartments, dry, well-ventilated chambers; he should make use of a full diet of nutritious, easily digested, and plainly-cooked food; the daily use of the bath, either tepid or warm, as the one or the other accords best with the feelings of the patient, followed by brisk friction of the entire surface of the body with a coarse towel or flesh-brush; and, finally, by the cultivation of a cheerful, buoyant, hopeful disposition. By the course here laid down, many individuals, with a strong predisposition to tuberculization of the lungs, may be saved much suffering, and their lives prolonged to an advanced period with comparative vigor and entire comfort.

